# csRNA and heparan sulfate : cell surface ribonucleoproteins regulate HS-mediated signaling

**DOI:** 10.1038/s41392-026-02727-z

**Published:** 2026-06-12

**Authors:** Jinlin Li, Jiaxin Ling

**Affiliations:** 1 Department of Medical Biochemistry and Microbiology, Uppsala University, Uppsala, Sweden; 2 Department of Medical Biochemistry and Microbiology, Zoonosis Science Center, Uppsala University, Uppsala, Sweden

**Keywords:** Molecular biology, Developmental biology

**Fig. 1:**
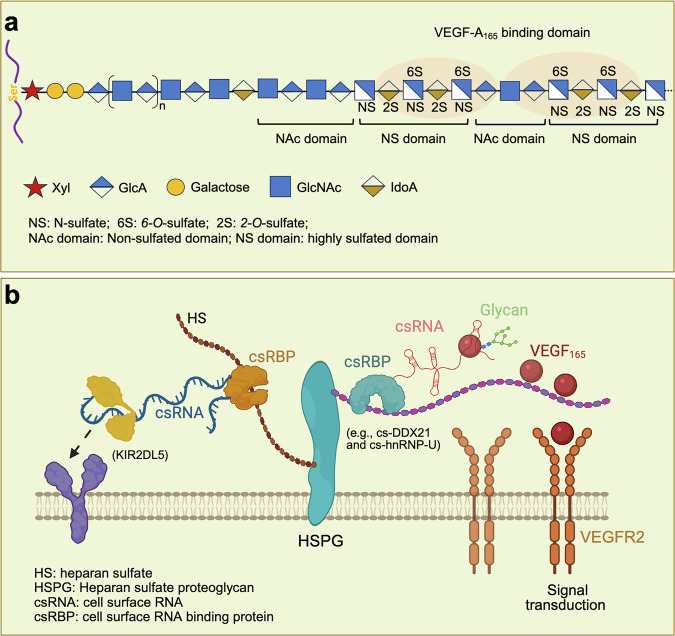
HS acts as a scaffold platform to regulate csRNAs' functions on the cell surface. **a** HS structure and domain organization. The modifications of its component sugars during biosynthesis (GlcNAc and GlcA or IdoA) result in the formation of distinct domains (such as VEGF_165_ binding domains, rich in sulfation), which are responsible for interacting with different factors. *6-O*-sulfated HS facilitates the cluster formation of HS, csRNAs, and csRBPs.^1^ **b** HS serves as a scaffold to form a complex with csRNAs (including GlycoRNA) and csRBPs (such as cs-DDX21 and cs-hnRNP-U). csRNAs complete the binding of HS to VEGF_165_, thereby regulating HS-mediated VEGF_165_ signal transduction.^1^ In addition to remodeling HS-mediated biological functions, the csRNAs attached to the HS scaffold can interact with other factors and influence cell signal transduction or other biological processes. As demonstrated in another recent research, csRNA is capable of recruiting the immune receptor killer cell immunoglobulin-like receptor 2DL5 (KIR2DL5).^4^ **b** of the figure was created in BioRender. Ling, J. (2026) https://biorender.com/pzsvz0z

In a recent study published in *Nature*, Peiyuan Chai et al.^1^ uncovered a new layer of regulation of cell surface ribonucleoproteins (csRNPs) in heparan sulfate (HS)-mediated VEGF-A signal transduction (Fig. 1). The discoveries not only provide a new understanding of csRNPs, including newly identified glycoRNA in important biological processes, but also open a distinct avenue to explore the detailed mechanisms of how HS precisely regulates various cell signaling pathways.

HS, a biopolymer ubiquitously expressed on the cell surface and in the extracellular matrix of almost all cells, is a member of the glycosaminoglycan (GAG) family, which consists of linear polysaccharides composed of repeating disaccharide units that can be modified by sulfate groups. The degree of sulfation and sulfation pattern mainly determine the HS-mediated biological functions through interacting with cytokines, growth factors, morphogens, cell-adhesion proteins, as well as other biological components, such as viral proteins. One of the classic examples is HS-mediated VEGF-A_165_ signal transduction, where HS can promote the formation of functional VEGFR complexes in *trans*.^2^ In addition to the differential expression and sulfation of HS, other mechanisms that modulate HS-mediated VEGF-A signaling are still ill-understood. Except for HS, RNA is another class of negatively charged biopolymers that can attach to the cell surface (cell surface RNAs (csRNAs)). A group of csRNAs has recently been identified as being modified with sialylated and fucosylated N-glycans, termed glycoRNAs.^3^ The functions of glycoRNAs and their interactions with other biopolymers on the cell surface remain to be investigated.

How glycoRNAs present on the cell surface remains not fully understood. Based on previous studies, which show that Siglec-11 binds to the cell surface in an RNA-dependent manner, the authors further revealed that only Siglec-11 out of 13 Siglec protein isoforms was sensitive to RNase treatment and was able to interact with sialic acid-modified glycoRNAs directly. To screen essential genes for organizing Siglec-11 and csRNA binding, the authors employed the genome-wide CRISPR-Cas9 gene-knockout (KO) method. Among the top hits of Siglec-11 screen, the HS biogenesis genes (EXT1, EXT2, and UXS1) were identified as the three most essential genes for Siglec-11 cell-surface binding. These three genes were also present among the hits from the 9D5 (an anti-dsRNA antibody that detects csRNA) screening. EXT1, EXT2, or UXS1 knocking-out (KO) cells completely lost both the Siglec-11 and 9D5 binding, whereas *EXT2* KO had no effect on Siglec-7 or Siglec-9 binding, suggesting HS chains are specifically required for the formation of the Siglec-11–csRNA cluster. A series of experiments in which the HS biosynthesis genes (EXT1, EXT2, NDST1, HS2ST1, or HS6ST1) were knocked out; alternatively, HS was removed from the cell surface by heparinase, or *6-O-*sulfation of intact HS was eliminated by overexpression of the two extracellular sulfatases, Sulf1 or Sulf2, demonstrated that intact HS chains with *6-O*-sulfation play critical roles in facilitating csRNPs clustering on the cell surface. The dependence of HS is not limited to Siglec-11 but also applies to other cell surface RNA-binding proteins (csRBPs), such as cs-DDX21 and cs-hnRNP-U (Fig. 1). Together, the results suggest that HS acts as a scaffold to tether csRNAs and csRBPs to the cell surface. The role of HS in orchestrating RNA and csRBPs is also pointed out in another independent study where csRNAs presented on the cell surface were shown to form complexes with RNA-binding proteins and HS to recruit immune receptors (Fig. 1b).^4^

HS has been known to regulate VEGF-A signal transduction by facilitating the binding of VEGF-A_165_ to its receptors. Accumulating evidence indicates that extracellular RNA (exRNA) may contribute to angiogenesis through modulating VEGF signaling.^5^ Given the scaffold role of HS in retaining csRNAs on the cell surface, the authors aimed to elucidate the potential impacts of csRNAs on VEGF-A-mediated signaling. RNase treatment caused a 2–3-fold increase in VEGF-A_165_-induced signal transduction by enhancing the binding of VEGF-A_165_ to the HS-VEGFR2 cluster on the cell surface. VEGF-A_165_ can interact with small RNAs in vitro and on the cell surface, which is further validated by the findings that R/K mutant VEGF-A_165_ (where all eight arginine residues in the VEGF-A_165_ HS domain are changed to lysines (HS(R/K)) attenuates its csRNAs binding ability while maintaining its HS binding capacity. VEGF-A_165_ HS(R/K) is able to recapitulate the enhancement of wild-type VEGF-A_165_ signal transduction resulting from RNase treatment. In addition, VEGF-A_165_ HS(R/K) failed to enrich sialoglycoRNA by RNA immunoprecipitation, whereas VEGF-A_165_ did. The small RNA treated with sialidase led to around a 35% reduction in its interaction with VEGF-A_165_ as assessed by a microscale thermophoresis assay. Altogether, the data indicate that HS, VEGF-A_165_, and csRNAs form a cluster on the cell surface, and csRNAs negatively regulate VEGF-A_165_-mediated signal activation by competing with its binding to HS. GlycoRNAs are among those VEGF-A_165_-bound csRNAs. To further explore if csRNAs regulate VEGF-A_165_ in vivo, the authors examined the effect of VEGF-A_165_ (R/K) on angiogenesis using neonatal mouse retina and zebrafish (*Danio rerio*) embryo models. The results demonstrate that VEGF-A_165_ (R/K) mutant enhances angiogenesis in both models.

Taken together, this study demonstrates that csRNAs serve as a negative regulator of VEGF-A_165_ signal transduction through cluster formation of HS and csRBPs on the cell surface, highlighting that csRNAs may play essential roles in other important biological processes. Even more importantly, the scaffold role of HS in tethering csRNPs uncovered in this study provides a new model to decipher functions of csRNAs, including HS- dependent and independent biological activities (Fig. 1b). There are still many interesting questions worth addressing. A relatively abundant amount of GlycoRNAs is among the csRNAs that can bind to VEGF-A_165._ What are the functions of glycan modification in csRNAs-mediated VEGF-A_165_ signal transduction? Additionally, it will be important to know if the ability of HS to function as an anchor platform is a common mechanism underscoring the regulatory roles of csRNPs. It also remains to be investigated how the interaction between HS and csRNPs orchestrates their functions.
